# She or He? Source of Errors in L2 Production of 3rd Person Singular Pronouns by Chinese Speakers of English

**DOI:** 10.1007/s10936-026-10226-z

**Published:** 2026-04-04

**Authors:** Junmin Li, Xin Wang, Qingli Zheng, Alessandro Giovanni Benati, Haiquan Huang, Junjie Wu

**Affiliations:** 1 Hangzhou City University, Hangzhou, China; 2 Department of Linguistics, Macquarie University, 16 University Ave, Sydney, Australia; 3 Department of Education, Oxford University, Oxford, UK; 4 Centre of Applied English Studies, University of Hong Kong, Hong Kong, China; 5 School of Education, University College Dublin, Dublin, Ireland; 6 School of Foreign Languages, Jianghan University, Wuhan, China; 7 Key Research Base of Humanities and Social Sciences of the Ministry of Education, Academy of Psychology and Behavior, Tianjin Normal University, Tianjin, China

**Keywords:** Chinese learners of English, Gender production, 3rd person singular pronouns, L2 speech production, Second Language Acquisition

## Abstract

A notorious problem in L2 English production is *he/she confusion* in 3rd person singular pronouns. Even proficient Chinese speakers of L2 English tend to misuse *he* for *she* in production, causing communication barrier regarding who is the antecedent. The present study investigates whether this processing problem in L2 production is associated with a few factors being investigated and provides practical solutions in L2 learning and teaching. We employed a story-listening-and-retelling production task to examine the effect of internal and external gender cues to discourse to elicit production by proficient Chinese speakers of L2 English. The results show an effect of gender complexity (i.e., internal cues) but no effect of picture (i.e., external visual cues), indicating gender information in L2 was computed at the conceptual level but not accurately processed for output in production. In addition, we found Age of Acquisition (AoA) effect in producing L2 pronouns without external cues. We discuss our findings in relevant speech production models and present pedagogical implications for EFL/ESL Chinese teachers and learners.

**Supplementary Information:**

The online version contains supplementary material available at 10.1007/s10936-026-10226-z.

## Introduction

Producing a second language imposes a number of challenges to L2 speakers whose L1 lacks gender marking (e.g., in third-person pronouns), among which L2 gender production remains difficult even for highly proficient learners(Alarcon, 2011; Bordag & Pechmann, 2007; Keating, 2009; McCarthy, 2008; Montrul et al., 2008; White et al., 2004). Corbett (1991) categorized languages into several different groups, regarding how gender information is encoded at the phonological, morphological and semantic levels, among which the prominent group has a formal/grammatical gender system, where all the nouns are assigned a gender (e.g., masculine or feminine in Spanish). Another group does not assign genders to nouns or verbs, but its pronouns present the *only* evidence for gender, having an agreement between the anaphoric pronouns and their antecedents (the girl…she). Corbett (1991, p5) named such systems ‘pronominal gender systems’ (e.g., Chinese or English). Most research on L2 gender production focuses on the sharp contrast between native speakers and L2 learners in their use of gendered nouns and in tasks involving pronouns of languages with grammatical gender systems (e.g. Bordag & Pechmann, 2007; Gruter et al., 2012). Less attention has been paid to L2 gender production in those languages of ‘pronominal gender systems.’ Languages of these systems do not mark gender but simply follow the gender agreement between the anaphoric pronouns and their antecedents.

In a language of pronominal gender systems, gender production is assumed to be simple and straightforward, as observed in native speakers. Though some complication can occur within L2 speakers due to the mismatched phonological encoding in L1 and L2. For example, both English and Chinese use a pronominal gender system, but they differ from each other in gender forms of third-person (3rd person) pronouns in speech (see Table 1). For the 3rd person nominative singular pronouns, English has *he* (masculine animate), *she* (feminine animate) and *it* (inanimate); but spoken Chinese uses *ta* for all three. In contrast, Chinese writing system does differentiate these three *ta* in 他 (he), 她 (she) and 它 (it), yet even ancient Chinese written form did not differentiate them.^1^

**Table 1:** A contrast of 3rd person singular pronominal systems in spoken Chinese and English

	Case	Chinese	English	
		#	Masculine	Feminine
3rd person	Nominative	*ta*	he	she
	Accusative	*ta*	him	her
	Possessive	*ta de*	his	her
	Reflexive	*taziji*	himself	herself

# No gender is distinguished in spoken Chinese

Moreover, while English has an obligatory subject and co-referential pronouns in declarative sentences, Chinese allows null topics and is a pro-drop language (Huang & Yang, 2024). This means pronouns are frequently omissible (and often more naturally omitted) in grammatical sentences. For example, the sentence “Zhangsan shuo bu renshi Lisi (Zhangsan say not know Lisi)” could mean “Zhangsan said he did not know Lisi” or “Zhangsan said [somebody else] did not know Lisi” (where the topic/somebody else is null) (Huang, 1984). English, however, typically requires overt pronouns. Therefore, the information needed to understand an English sentence is usually explicitly present within its structure (e.g., Huang, 1984; Li, 2007). Thus, gender information in Chinese is rather minimally represented at the surface level (e.g., in pronouns), even compared to English, but somehow encoded in the discourse.

The fact that English marks the distinction between the masculine and feminine on 3rd person singular pronouns while Chinese does not, appears to impose production difficulty in native Chinese speakers of English. Some studies have reported the learning difficulty of 3rd person pronouns of English by Chinese learners (Chai, 2010; Dong & Jia, 2011; Liu, 2007). Dong and Jia (2011) identified a noteworthy discrepancy in error rates related to English gendered pronouns among Chinese learners. They reported an average error rate of 6.47% based on a corpus survey, which included data from second-year English majors at a Chinese university. This rate escalated to 15% in a Chinese-English interpretation task conducted with proficient English learners. These findings underscore the persistent difficulty advanced Chinese learners encounter in mastering gender-specific pronoun usage in L2 English.

The issue of gender errors among Chinese learners of English appears to be distinct from that observed in other learner groups as documented in the literature. For example, the error rates shown in Dong and Jia (2011) substantially exceed those found in studies by Antón-Méndez (2010, 2011) involving L2 English learners of L1 backgrounds in Dutch, Spanish, and Italian. This suggests that the nature and degree of persistence of gender errors in L2 acquisition may vary considerably across different linguistic backgrounds and profiles. Analyzing data from the Louvain International Database of Spoken English Interlanguage (LINDSEI) corpus, Chen (2004) discovered that the gender error rate among Chinese learners of English was notably high at 17.65%. This rate was significantly higher compared to that of Japanese and French EFL learners, whose native languages do make gender distinctions in 3rd person pronouns, with respective error rates of 4.2% and 0.92%. The impact of the Chinese language background on English learning appears to be more pronounced and enduring than that of other languages.

The existing literature on L2 gender errors by Chinese speakers is relatively scarce. Three studies led by Dong and her colleagues show that gender information in 3rd person singulars was not processed the same way by L2 speakers as L1 speakers. First, Dong and Jia (2011) analyzed error rates in various grammatical cases of 3rd person singular pronouns in both corpus survey and interpreting task performed by Chinese learners of English. They observed a higher frequency of gender errors in the nominative and possessive cases than of those in the accusative and reflexive cases. The authors argued that the nominative and possessive cases required less attention and monitoring in L2 production given their more salient roles in the discourse, compared to the accusative and reflexive cases, thus generating more errors. Dong and Li (2011) further investigated the issue using self-paced reading to compare the reading times of *he* or *she* that matched or mismatched its respective antecedent, by manipulating the gender prominence of the antecedents either in a celebrity name condition (e.g., Bill Cliton or Gong Li, a well-known actress) or in a career name condition that encodes prototype/conventional gender (e.g., nurse or plumber). The study did not find a mismatch effect (i.e., increased reading times for pronouns that mismatched the gender of their antecedents compared to those that matched) in either condition. In contrast, such mismatch effects have been found in native English speakers (Carreiras et al., 1996; Kennison & Trofe, 2003; Duffy & Keir, 2004; Dillon et al., 2013; Sturt, 2003). Further analysis found a mismatch effect only for the female celebrity names. Post-experiment questionnaires indicated that participants perceived the gender of female celebrity names as more salient than that of male celebrity names or career names. Therefore, both studies suggest that implicit attention to gender information encoded in the discourse plays a role in L2 gender production. In addition, Dong et al. (2015) conducted two self-paced reading experiments (*N* = 66 and *N* = 32) to examine whether visual cues (human images) could enhance L2 Chinese-English speakers’ sensitivity to pronoun-antecedent gender agreement. Their study specifically investigated if correct gender matches (e.g., female antecedent + “she”) elicited faster reading times than mismatches, mirroring patterns observed in native English speakers. The findings showed an interaction between visual cue and gender match/mismatch; that is, the gender mismatch effect only appeared when the antecedent gender information was enhanced by an image. Given these findings, Dong et al. proposed that the frequent interchangeability (i.e., confusion) of *he* and *she* in their speech could be attributed to inadequate processing of gender information at the conceptual level, which was not fully automatized in L2 speakers.

Collectively, these studies reviewed here indicate that L2 English pronoun gender errors stem at the conceptual level but warrants further investigation. Note that the abovementioned experimental studies employed self-paced reading, informing about *language comprehension*, it is unclear whether similar effects can be observed in *language production*, namely, in a task where the message (sentences) was *constructed initially* at the conceptual level given external prompts to better simulate daily communications (rather than a task where the message was given initially like in a translation/interpretation task). Language production is proactive, while language comprehension is reactive. Therefore, they engage in distinct cognitive processes. The key difference between the two is that production necessitates both grammatical and phonological encoding prior to articulation, thus, more error-prone than comprehension. We will return to the details of this in the following section.

### Cognitive Mechanisms in Language Production

According to Levelt’s (1989, 1995) language production model, the speech production process is divided into four steps: message generation, grammatical encoding, phonological encoding, and articulation. Prior to articulating a *message*, the speaker engages in the preverbal phase, which entails selecting lexical units from the mental lexicon. During this stage, the speaker references components of the conceptual framework to extract suitable words. Speakers formulate the structural foundation of their speech without primary consideration of the phonological aspects of the words to be produced. The inter-layer between the preverbal and phonological encoding is referred to ‘lemma’. When a speaker has retrieved a lemma, it implies that the speaker has gained access to the components of the word’s stored information that are pertinent for creating the syntactic context of the utterance. Within the lemmas of our mental lexicon, conceptual information is interconnected with its grammatical role.

Processing pronouns requires the identification of a mental referent, which is the concept or object the pronoun stands for, as well as a discourse antecedent (if there is, which is the preceding word or phrase the pronoun refers to in a spoken or written context). One of the key indicators of the connection between a pronoun and its antecedent is the pronoun’s agreement in grammatical features, such as being singular or plural (number) and matching the gender of the antecedent. This agreement is a clear and direct signal that helps to establish the relationship between the pronoun and the noun it represents. Research by Cloitre and Bever (1988) and Gernsbacher (1991) suggests that the mental representation of a pronoun’s antecedent closely resembles a referential, model-based structure. This mental model helps to create a coherent and connected representation, aiding comprehension. This form of representation is based on conceptual understanding rather than surface-level linguistic forms. It suggests that when individuals process pronouns, they do so by linking them to a mental model that captures the underlying meaning of the antecedent, not merely its grammatical or syntactic features.

Regarding L2 production, most work focuses on whether and to what extent L1 and L2 influence each other. Some show that gender information was automatically accessed in production, as in the automatic gender-access models (Caramazza et al., 2001; Schiller & Caramazza, 2002; Costa et al., 2003). That is, the gender of a noun in L2 is automatically available as soon as the noun’s lexical node is selected. In contrast, others believe that L2 speakers don’t initially limit their search to the L2 lexicon. Instead, both L1 and L2 lexicons are activated and searched at the same time (De Bot, 1992; Hermans et al., 1998; Grosjean, 1998). It is widely accepted that the L1 and L2 systems interact at both conceptual and phonological levels (De Groot, 1992; De Groot et al., 1994; Costa et al., 2000).

Furthermore, Bordag and Pechmann (2007) argued that gender information in L2 is not stored as a fixed feature for each lemma but is computed real time as required, based on all available information, including stored information from previous inputs and competition results. Thus, gender access in L2 is not a direct or automatic result of lexical selection, but subject to L1 influence. Their results support that L1 and L2 systems interact not only at the conceptual and phonological level, but also in grammatical encoding at the lemma level specified in Levelt’s model (Levelt, 1989; Levelt et al., 1999)^2^.

### Factors in L2 Production

Historically, the age of acquisition (AoA) effect on lexical development and processing has been extensively discussed in L2 literature, primarily as a result of neural plasticity (Abutalebi, 2008; Hernandez et al., 2005; Li et al., 2014; Wartenburger et al., 2003). According to this perspective, when L2 learning takes place early in life, the L1 system is less fixed, allowing brain regions responsible for L1 processing to remain flexible enough to support the acquisition of the L2 system. In word production studies, the AoA refers to the phenomenon that words acquired earlier are processed more quickly and more accurately than words acquired later in word production (Rochford and Williams 1962a, b).

However, in a study by Foote (2010), the production of subject-verb number agreement was examined among early English-Spanish and late Spanish-English bilinguals with various levels of proficiency. The findings indicated that proficiency levels significantly influenced the performance on agreement tasks, while L2 AoA did not appear to affect the fundamental processes underlying agreement production. However, these two factors are closely linked, namely, those who learned a language earlier also showed higher proficiency (e.g., Hernandez, 2013; Li, 2013; Monney et al., 2013). Despite this, Berghoff et al. (2021) found no correlation between AoA and proficiency in L2 lexical processing. Both L2 proficiency and AoA independently influence cross-language activation.

Another important source of L2 gender errors, investigated and argued by Antón-Méndez (2010, 2011), is attributed to whether L1 is pro-drop or not. The author tested Spanish and French speakers of L2 English on he/she confusion errors, who had comparable L2 proficiency and AoA. The results showed that Spanish participants produced significantly more gender confusion errors (he-error rate at 2.98% and she-error rate at 5.68%) than their French counterparts (0.42% for he-error and 0.92% for she-error). Antón-Méndez reasoned that L1 Spanish speakers do not need to encode gender information when preparing a preverbal message because Spanish is a pro-drop language but both English and French require the encoding of gender information for a preverbal message as a non-pro-drop language. This discrepancy between Spanish and English pronoun production resulted in higher error rates in Spanish speakers than French speakers of L2 English. Antón-Méndez (2011) further investigated gender errors in the production of English possessives (i.e., “his” and “her”) by native Spanish, Dutch and Italian speakers of lower advanced level proficiency in L2 English. The results showed that proficient L2 speakers were likely to be influenced by their L1 and suggested that a speaker would automatically implement a certain L1 procedure in their L2 processing. Thus, Antón-Méndez concluded that these gender errors in L2 pronoun use were caused by differences in how L1 and L2 pronouns are co-referenced in discourse.

Finally, some reports suggested the use of a default gender strategy among L2 speakers. That is, L2 speakers tend to assign the more frequent gender to a particular noun or object: for example, the common gender in L2 Dutch (Sabourin et al., 2006) and the masculine in L2 Spanish (White et al., 2004). Default forms may refer to the most frequent morphophonological forms within a paradigm, the forms that are unmarked for gender or number features, or the combination of both (Tsimpli & Hulk, 2013).

In summary, research on the acquisition and processing of L2 gender has focused on various factors, including the AoA effect, L2 proficiency, discrepancies in the gender marking systems between L1 and L2, and L1 morpho-syntactic properties.

### Pronoun Gender Production in Chinese Learners of English

In the case of Chinese learners of English, first, Chinese is a subject-drop language, which may influence L2 gender production as discussed earlier. Second, both British National Corpus (BNC) and the Modern Chinese Corpus (MCC) show that the masculine pronouns (English he; Chinese 他/ta/) significantly outnumber the feminine ones (English she; Chinese 她/ta/). The BNC shows a near 2:1 ratio, while the MCC indicates a ratio exceeding 2:1 (Table 2). Third, the/h/in ‘he’ is acquired earlier and articulated more easily than the/ʃ/in ‘she’ by English native speakers. This difficulty might be compounded for Mandarin speakers, as/ʃ/is absent from the Chinese phonemic inventory. Fourth, at the morpho-phonological level, English pronouns act as a reliable morphophonological indicator of biological gender, outweighing the importance of semantic cues. Thus, morphophonological and semantic cues align and reinforce each other. Conversely, in spoken Chinese, pronouns lack a morpho-phonological cue to specify the gender of the antecedent they refer to, such that the semantic cue stands alone as the sole guide for determining gender. The gender errors in L2 pronoun production could also be driven by this cross-linguistic contrast.

**Table 2:** Frequency of *he*/他 and *she*/她 in BNC and MCC

Corpus	he/他frequency	she/她 frequency
BNC#	639,449	351,579
MCC##	52,912	19,915

# a total of 100 million words

## a total of 20 million characters

Given above, L1 Chinese, as a subject-drop language without any morph-phonological gender cues, is more likely to negatively impact the processing of 3rd person pronoun in L2 English. Our approach to this problem is to investigate L2 speakers’ sensitivity to gender cues in various contexts in order to trace the sources of L2 errors. To achieve this, we manipulated gender cues at two different levels: external and internal. Here, external cues refer to information external to oral discourse but meaningfully indicating the gender of agents. Pictures served as the external cues to enhance the gender information of agents (with vs. without pictures). The internal cues, encoded in the names, kinship terms, and stories, provide gender complexity (single gender vs. mixed gender). We aim to answer four research questions in a story-listening-and-retelling production task.

RQ 1: Do external gender cues, represented by pictures to facilitate L2 production, affect the error rates of masculine and feminine 3rd person pronouns?

RQ 2: Do discourse gender cues (internal), represented by gender complexity in discourse, affect the error rates of masculine and feminine 3rd person pronouns in L2 production?

RQ 3: Given the higher frequency of *he* in both English and Chinese, is the masculine the default gender used by Chinese learners of English?

RQ 4: Are AoA and L2 proficiency relevant factors when producing 3rd person singular forms in Chinese learners of English?

To optimize our goal, instead of using a comprehension task, we asked participants to listen to a short story involving two agents and then verbally produce it, to authentically assess their production in L2 gender. We recruited advanced English learners to ensure meaningful evaluation of their performance. The study manipulated two distinct gender cues (internal and external), though we did not examine their interaction. Instead, our design focused on their independent effects on L2 gender production among Chinese speakers. AoA and L2 proficiency effects are complementary in our investigation, as they are not controlled in the study given the sample size.

## Methods

### Participants

Thirty-two native Chinese speakers (9 males and 23 females, mean age = 24) studying at a University in the UK were paid to participate in the experiment. Table 3 presents the background of the participants, as well as their IELTS scores. Twenty-five participants achieved a score of at least 7 (Skill Level: Good User) out of 9 (Skill Level: Expert User) in the IELTS, among whom six studied full-time in English-as-the-medium-of-instruction contexts for the last two years prior to testing. The rest seven attained an equivalent IELTS score converted from the TOEFL iBT scores using official tools developed by the ETS. All participants were native Chinese speakers, reporting having learned English through formal classroom instruction in China prior to their UK residence. During their time in the UK, they reported using English in approximately 85% of their daily communication contexts. All participants signed the consent forms for the present study approved by the Departmental Research Ethics Committee (DREC) after reading the information sheet concerning the study. The participants were also asked to fill out a Language History Questionnaire (Appendix A in Supplementary Material S2) prior to the experiment.

**Table 3:** Descriptive information of the participants

	Minimum	Maximum	Mean (SD)
Self-rated Speaking*	3	7	5 (0.80)
IELTS Band Score	6	9	7.43 (0.69)
Age of Acquisition	3	14	9.59 (2.77)
Length of Residence	0.50	10.00	3.25 (2.02)

*Self-rated speaking scores were given on a 7-point Likert scale, where 7 is “native-like” and 1 is “very poor”

### Materials and Design

Our main approach to the study design is to present participants stories and discourses to elicit their production in L2 in a natural and straightforward way, without biasing them towards a particular linguistic structure we are testing. Thus, we modified 32 simple stories from New Headway Intermediate (4th Edition) by Oxford University Press. Each story was then proofread and revised by a native English speaker to ensure its accuracy and naturalness. Every story contained 2–4 simple sentences involving two agents. Between the two agents, one was the major and the other the minor. The major (bolded in the example given in Table 4) narrated the story, while the minor served the role to create the gender condition.

**Table 4:** Three conditions for 3rd person singular pronoun production

Conditions	Specification	Examples
Single Gender Condition	Stories include either two females (*n* = 8 items) or two males (*n* = 8 items) as agents	Single (two female or male agents): 1) **Sarah** and Sophie “My friend Sophie will get married next month. I’m very excited. Sophie has sent me an invitation for the wedding.”
		2)**William** and father I finished all the courses for my degree. My father is very proud of me. He’s going to host a party to celebrate with me.
MIXED Gender Condition	Stories include one female and one male as agents (*n* = 8 items)	MIXED (a female/male major agent + a male/female minor agent): 3) **Sarah** and Peter: “My friend Peter will get married next month. I’m very excited. Peter has sent me an invitation for the wedding.” 4)**William** and wife I finished all the courses for my degree. My wife is very proud of me. She’s going to host a party to celebrate with me.
NoP Condition	Stories include either two females (*n* = 4 items) or two males (*n* = 4 items) as agents	NoP (two female or male agents without picture cues): 5) **Sarah** and Sophie: “My friend Sophie will get married next month. I’m very excited. Sophie has sent me an invitation for the wedding.” 6)**William** and father I finished all the courses for my degree. My father is very proud of me. He’s going to host a party to celebrate with me.

This study adopted a within-participant and within-item design, with two independent variables as *gender complexity* (two levels: Single Gender vs. MIXED Gender) and *picture cues* (two levels: with Picture vs. NoP) and one dependent variable as the *error rates* of the production of 3rd person singular pronouns in English. The mixed gender condition was more complex because two referents were of different genders, both ‘she’ and ‘he’ were activated and competed for the speaker’s attention, unlike in the single gender condition, where both referents were either females or males. Considering the item number and the theoretical interests of these two independent variables, we only included Single Gender in items without picture support. In other words, we were not interested in the interaction between *gender complexity* and *picture cues*, thus, the condition without picture was based on single-gender stories.

**Fig. 1:**
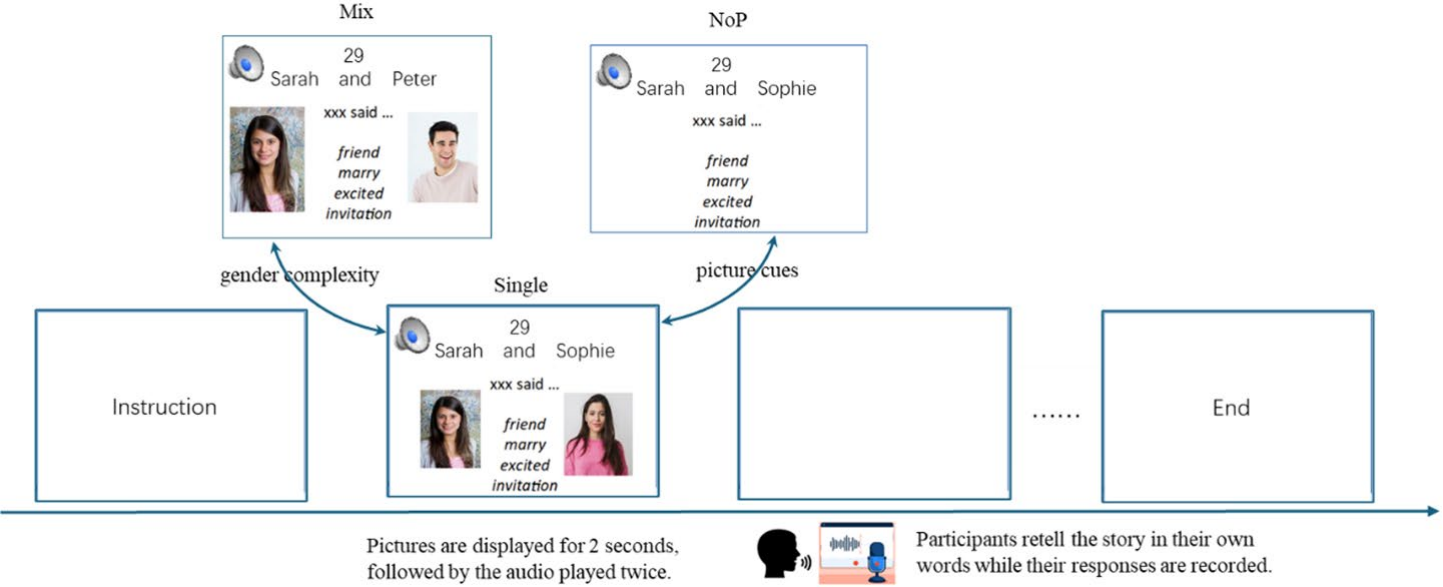
Schematic overview of the task procedure in different conditions

A counter-balanced design was adopted such that each participant was tested with all test conditions and each item (story) was tested in all condition, as illustrated in Table 4; Fig. 1, resulting in 4 counterbalanced lists. Across the four lists, only the names of the two agents differed; other information remained unchanged. Each name was used only once within each list to avoid repetition. In the conditions with the picture cues that indicate the two agents, the visual information clearly specified the gender complexity in addition to the names or kinship terms (i.e., whether both agents were males or females or one male and one female). In the conditions without the picture cues, gender complexity was not a variable as stated above, and thus not coded. All items were either single-male or single-female stories based on names and kinship terms, except for a few items which could be gender-neutral which we removed in analyses. Importantly, we chose names which were most unambiguous in gender, to represent the two agents in each story such that participants could figure out the gender information from the two agents’ names.

For the names of the major agents, 22 female names and 22 male names were chosen from the *Top 100 baby names for each gender in English and Wales*,* 2011* on the guardian website; the rest were presented in a “Mr./Miss/Mrs + surname” pattern with the surnames selected from the internet sources. The minor agent was either given a common name (from the same source, but different from that of the major agent) or assigned a relationship with the major agent, like mother, father, roommate (normally considered of the same gender), etc. Appendix B in the Supplementary Materials S2 provides 32 stories in each condition.

Importantly, we played each auditory story to participants in either a female or male voice as the narrator, which gave additional cue for gender. All the stories were recorded by a male and a female native English speaker, via the open-source software Audacity, version 2.0.5 (Audacity Development Team, 2013). For the mixed condition, there was a female voice when the major agent was female, and male when the major agent was male. All recordings were normalized. In addition, the pictures used were first chosen from proper online galleries of real people accessed via Google Images and then double checked by the second author.

### Procedure

The participants were randomly assigned to one of the four lists. They were presented with both auditory and visual stimuli through Power Point slides on a MacBook Pro. Their responses were recorded on an iPhone 4 handset either in a quiet room or a silent booth.

During the presentation of each trial (see Fig. 1), an audio icon was present in the upper-left corner. Once clicked upon, it played the story recorded for the experiment. In the upper-center position were the names of two agents; as the narrator of the story, the major agent was always on the left. Below the names was a response template for participants—“xxx said …”. “xxx” was the major agent and the narrator of the story. Keywords of the story (usually four words taken from the script, mainly nouns, verbs, adverbs, or adjectives) were listed just below the template to reduce the workload of working memory. For the picture conditions, the pictures of the agents in the story were presented on each side of the keywords, as shown in the sample trial in Fig. 1. For the NoP condition, no pictures of the agents were provided. Other than the four test lists, a practice list of 4 trials, different from the test trials, was created following the same procedure and presented to participants before the actual experiment. Prior to the practice, participants were provided with instructions and aware of the structure of each story (i.e., the major and minor). When they retold the story, they needed to use 3rd person singular pronouns to refer to the major who narrated the story.

When participants were instructed in front of the computer, for every trial, the names and pictures of the two agents (if in a without-picture condition, only the names) were first presented on the screen and stayed for about two seconds to allow the participants to process information from both names and pictures. Then the audio was played, together with the presentation of the keywords, so that the participants could register auditory information along with the keywords/prompts. The audio was played twice in total. Right afterwards, the participants were instructed to retell the story following the template “xxx said …” on the screen. Participants’ voices were recorded throughout the experiment. A sample answer to Fig. 1 trial would be:

***Sarah*** *said* ***her*** *friend Peter would get married next month.* ***She*** *was excited. She also said Peter had sent* ***her*** *an invitation …*.

After the experiment, none reported clear awareness of the research purpose, with many of them speculating that the experiment might examine some issues about tense marking.

### Scoring and Coding

Participants’ 3rd person singular pronouns were extracted and coded in ELAN 4.6.2 (http://tla.mpi.nl/tools/tla-tools/elan/*).* Six tiers were created based on the six categories of pronoun production defined in the Coding book (see Table 5). Each pronoun produced was marked and noted in the corresponding tier trial by trial, participant by participant.

**Table 5:** Coding conventions for target pronouns

Codes	Specification
N of HE	Number of occurrences of 3rd person masculine pronouns, i.e., he, his, him, himself, used correctly without self-correction; (the four pronouns will be interchangeably referred to as “he” pronouns in the paper)
N of SHE	Number of occurrences of 3rd person feminine pronouns, i.e., she, her (possessive), her’ (accusative), herself, used correctly without self-correction (these four pronouns will be interchangeably referred to as “she” pronouns in the paper)
N of HE Errors	A “HE-Error” is defined as a case where a masculine pronoun should be used, but a feminine pronoun was used instead.
N of SHE Errors	A “SHE-Error” is defined as a case where a feminine pronoun should be used, but a masculine pronoun was used instead.
N of HE Corrections	A “HE-Correction” is defined as a case in which a masculine pronoun should be used, but a feminine pronoun was produced first, and then self-corrected to the right form.
N of SHE Corrections	A “SHE-Correction” is defined as a case in which a feminine pronoun should be used, but a masculine pronoun was produced first, and then self-corrected it to the right form.

As for coding for production errors, a feminine pronoun error, coded as a *SHE-error*, refers to the case in which a feminine pronoun should have been produced, but a masculine form was produced instead. Similarly, a masculine pronoun error, coded as a *HE-error*, indicates that a feminine pronoun is mistakenly produced when a masculine one should have been produced. In addition, there were other criteria established to serve the purpose of the study: (a) repetitions were counted as 1 occurrence (e.g., one occurrence of N of HE was counted from the utterance “*he … ur*,* he… he was excited”*); (b) pronouns correct in gender but wrong in case were considered correct occurrences since case is a syntactic feature instead of a conceptual one (Bock & Levelt, 1994) (e.g., *he* from the utterance “*he uncle*” was calculated as 1 occurrence of N of HE); (c) if the participant only partially articulated a pronoun of the wrong gender before making the right choice (e.g., on the articulation of a/h/sound, an immediate change to the correct choice/ʃiː/*“she”* was considered as 1 SHE-Correction), this was considered correct; (d) in single-sex conditions, a pronoun correct in gender was deemed as one occurrence though the referent was semantically unclear (e.g., in the utterance “*David said every night he watched the star with Bob*,* and* *he* *wishes someone could get a star …*”, the underlined *he* was treated as one occurrence of N of HE, although it was not clear whether *he* referred to *David* or *Bob*).

Two authors/raters reviewed the context in all conditions to verify which character was being referenced in the recorded utterances, ensuring accurate coding. As seven trials in the NoP condition included ambiguous gender information such as “friend”, “roommate”, they were excluded from analysis. Our dataset and R codes are here: https://osf.io/zbjx7/?view_only=7d9a68961cc6407eb80982efc8d11a06.

## Results

The final dataset coding the accuracy was based on each attempt of pronoun production as a binary response (correct or not) per participant, instead of the accuracy rate per item per participant. We also examined each participant’s accuracy rate and excluded one outlier (below 65%) in the final analysis. Three items from two participants were not coded in the dataset because the participants failed to produce much utterance on the three items. Considering the amount of self-correction rate on average at the group level, which is roughly 4%, we decided not to include self-correction data in the analyses, which could complicate data interpretation, such that we could have a more accurate measurement of L2 pronoun production.

The descriptive statistics of *HE/SHE*-error rates under different conditions were presented in Tables 6 and 7, respectively. Given our design and research questions (i.e., we investigated the effect of gender complexity or the role of visual cues in 3rd person gender production), the meaningful comparisons should be based on accuracy rate calculations of HE or SHE produced in conditions where stories were presented by either Single Gender vs. Mixed Gender condition, or with Pictures vs. without Pictures. In other words, our comparisons were based on accuracy rates produced in conditions which only differed by one variable to demonstrate the effect produced by that variable (i.e., Mixed vs. Single; Picture vs. NoP). Within each variable, there were two levels (Female vs. Male). Given our design and theoretical interests on the separate effects of internal and external gender cues, we ran two models separately: (1) on the effect of gender complexity; (2) on the effects of picture cues. The first was conducted only on the picture-present conditions (Single vs. Mixed), while the latter was conducted only on the single-gender conditions (with vs. without pictures).

**Table 6:** Accuracy rates (%) in mixed and single conditions (SD in parenthesis)

	Mixed	Single
He	88.1 (0.10)	94.8 (0.06)
She	89.2 (0.11)	93.6 (0.08)

**Table 7:** Accuracy rates (%) in NoP and YesP conditions (SD in parenthesis)

	NoP	YesP
He	94.4 (0.10)	94.8 (0.06)
She	93.1 (0.09)	93.6 (0.08)

NoP: without pictures; YesP: with pictures

### Internal Discourse Cues of Gender Complexity

Statistical analyses on accuracy employed generalized mixed-effects models (Baayen, 2008; Baayen et al., 2008) with the lme4 package in the R environment (version 3.1.0; CRAN project; The R Foundation for Statistical Computing, 2008; Bates et al., 2015). We began with comprehensive models that included fixed-effects and the maximal random-effect structures (Barr et al., 2013) in a 2 (Mixed vs. Single) x 2 (He vs. She) design and then removed random slopes in a step-wise manner until the model converged. The fixed-effect factors were Gender Complexity (coded as *CueContext*: Mixed [0.5] vs. Single [−0.5]) and Gender (coded as *Pronoun*: he [0.5] vs. she [−0.5]). The final model included random intercepts for both items and participants to account for baseline variability in accuracy, as well as a random slope of Gender for items as follows:

glmer (ACC ~ CueContext * Pronoun + (1|SubNo) + (Pronoun + 1|ItemNo)

This structure accommodates item-specific differences in both overall performance and sensitivity to Gender, ensuring that the model captures the hierarchical and variable nature of the data. The model output showed a main effect of Gender Complexity (*β* = −0.83, *SE* = 0.14, *z* = −5.90, *p* < 0.001), without a main effect of Gender (*β* = −0.07, *SE* = 0.15, *z* = 0.46, *p* = 0.645) nor interaction *(β* = −0.29, *SE* = 0.30, *z* = −0.96, *p* = 0.337). Thus, only Gender Complexity showed a significant effect in producing errors in 3rd person singular pronouns in advanced Chinese learners of English. That is, more errors were observed in the mixed gender condition.

### External Cues of Pictures

Next, following the same procedure as in 3.1, we examined the effect of picture cues on gender production of 3rd person single pronoun in a 2 (with vs. without pictures) x 2 (he vs. she) design. Similarly, here, the fixed-effect factors were Picture and Gender. The final model was a simple one:

glmer(ACC ~ Picture * Pronoun + (1|SubNo) + (1|ItemNo))

The model output showed no main effects for either Picture (*z* = 0.87, *p* = 0.383) or Gender (*z* = 1.28, *p* = 0.200), as well as no interaction (*β* = −0.03, *SE* = 0.39, *z* = −0.07, *p* = 0.944). The non-significant effect indicates that picture cues were not an important factor in 3rd person singular pronoun production for the recruited second language population in our study. In addition, there was no difference between *he* and *she* in production.

### Correlation Between Overall Performance and AoA, and IELTS Scores

A linear regression model was performed to test whether AoA and/or L2 proficiency could best predict advanced L2 learners’ overall performance. Among the self-reported data summarized in Table 3, AoA and L2 proficiency were the widely discussed factors in the literature, therefore, we ran analyses on these two measures whose data were transformed to better fit the model. Across the whole dataset, there was neither effect for AoA (*β* = −0.01, *SE* = 0.01, *t* =−1.28, *p* = 0.213) nor effect for L2 proficiency (*β* = 0.01, *SE* = 0.01, *t* =−1.14, *p* = 0.267).

**Fig. 2:**
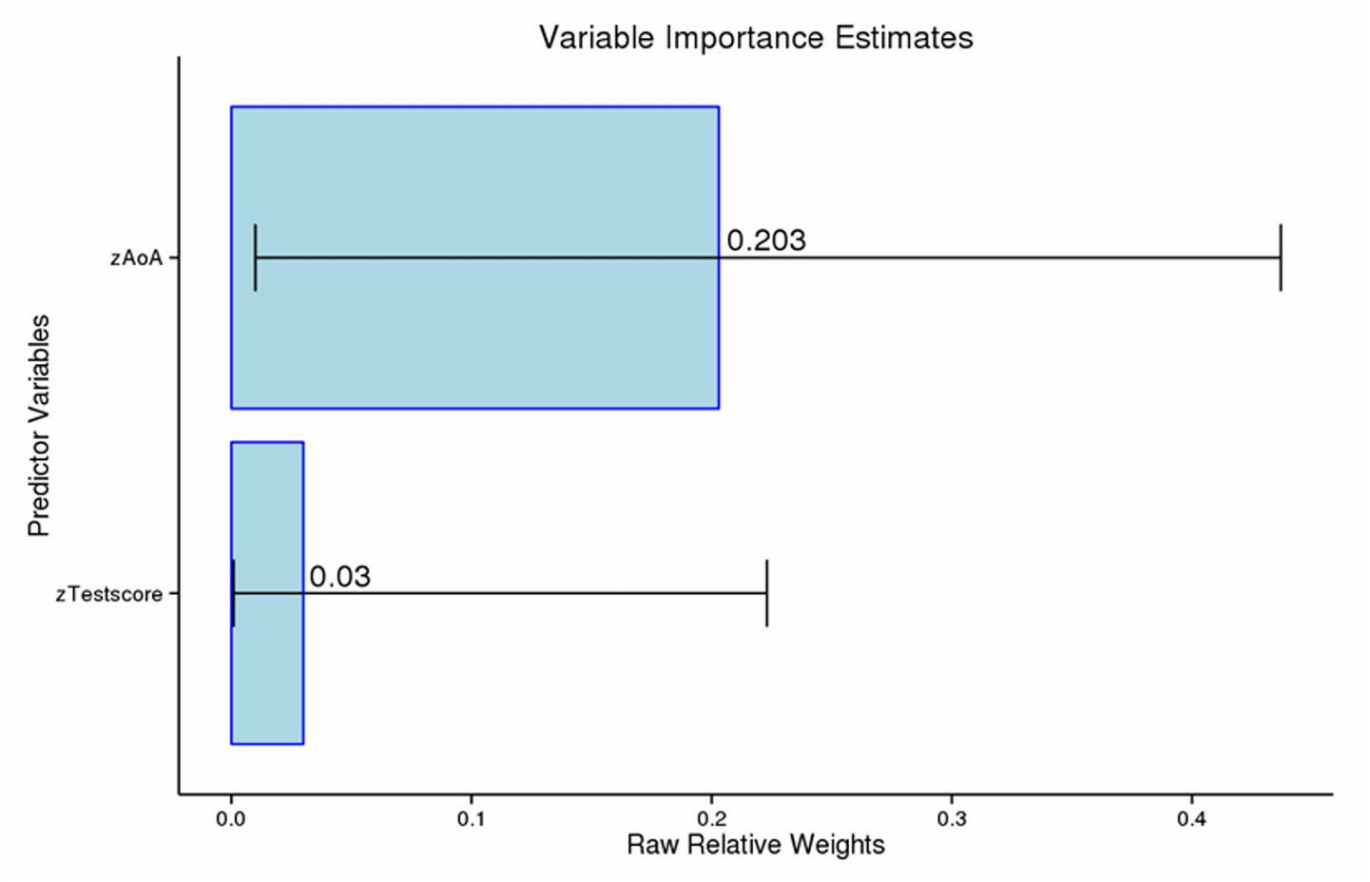
Dominance weights and corresponding 95% confidence intervals (R-squared = 0.23)

Given our particular interest in NoP trials, which simulate naturalistic discourse where speakers refer to absent third parties without visual support, we examined whether AoA and/or L2 proficiency effectively predicted performance in this context. We only found a significant effect of AoA for the NoP trials (*β* = −0.03, *SE* = 0.01, *t* = −2.53, *p* = 0.018). Although this analysis was theoretically motivated by our focus on discourse without visual cues, we also report the Bonferroni-corrected *p*-value (*p* = 0.053) to account for multiple comparisons, including two additional tests from the YesP trials in the Mixed and Single Gender conditions. This correction renders the effect marginal and is reported for transparency. Furthermore, to assess the unique contribution of each of the two predictors, we conducted dominance analysis and random forest modelling (Mizumoto, 2023, see Supplementary Material 3). The results confirmed that AoA influenced the overall accuracy rates only for those NoP trials, accounting for higher dominance weight (0.203 or 87.06%) in determining gender production accuracy than that of L2 proficiency (0.03, or 12.94%). Figure 2 displays the dominance weights and their corresponding 95% confidence intervals, arranged in descending order from the high dominance weight (i.e., AoA) to the low (i.e., L2 proficiency/Test Score).

For the trials with picture cues, separate linear regression models were also fitted for Mixed or Single Gender conditions, and no significant effects were observed for AoA (all *p*s > 0.299) or Test Score (all *p*s > 0.151).

## Discussion

To summarize, our study employed a story-listening-and-retelling task to investigate the factors relevant to the 3rd person singular pronouns production in proficient Chinese(L1)-English (L2) speakers. We found: (1) the mixed gender conditions generated more errors than the single gender conditions, indicating that gender information of the antecedents was computed at the conceptual level during the 3rd person singular pronoun production; (2) unlike the discourse cues, the null effect of picture cues suggest that L2 learners were not sensitive to external cues of gender. Based on these two sets of evidence, Chinese speakers do not appear to have difficulty encoding the gender information at the conceptual level when speaking L2 English; (3) the error patterns in production were not consistent with those in previous reports (e.g., Dong et al., 2015), showing that there was no difference in producing *she* or *he* errors. This evidence indicates that Chinese speakers of L2 English do not adopt a default gender strategy in production, showing no preference of the masculine forms over the feminine forms as comprehension task revealed. (4) AoA predicted accuracy in gender production only in stories without pictures, indicating that earlier AoA was associated with better production performance with the absence of external cues.

First, our findings provide evidence for the conceptual level computation of gender in L2 production of 3rd person singular pronouns, lending direct support to Antón-Méndez (2010) and Dong et al.’s (2015) findings and arguments. To be more specific, the effect of gender complexity reveals that advanced L2 learners were fully aware of the mapping between *he/she* and their related gender cues, but somehow the computed gender information was not accurately processed for output. This discrepancy between the knowledge at the conceptual level and processing might be largely due to the transfer effect from L1 Chinese, which is a subject drop language and prefers zero anaphora. As discussed earlier, a zero anaphora does not require the encoding of gender in production (i.e., no need to produce the antecedent), like in Spanish, but English is quite the opposite (Anton-Mendez, 2010, 2011).

To complicate this more, Chinese in speech does not mark the distinction between the masculine and feminine 3rd person pronouns. This influence from L1 adds additional difficulty in L2 gender production of 3rd person pronouns. We can reasonably assume that L2 learners, influenced by L1 Chinese pro-drop structures, dropped pronouns in English. As a result, when speaking in English, they struggle to determine which pronoun (*he/she*) to use in a given context. This difficulty aligns with Huang and Yang’s (2024) observation that in Chinese, a sentence like “Zhangsan shuo hui lai” (literally “Zhangsan said would come”) is grammatically complete and interpreted as “Zhangsan said [he/Zhangsan] would come”. The absence of an overt pronoun—permissible in Chinese due to its pro-drop nature—demonstrates how native speakers of such languages may not habitually encode pronoun gender, leading to challenges when selecting he/she in L2 English, where pronouns are obligatory. Furthermore, the optional nature of gender marking in Chinese pronouns (i.e. the unmarked status of 他) may contribute to reduced sensitivity to obligatory gender distinctions in English, explaining persistent pronoun errors even in contexts where gender reference is transparent.

Bordag and Pechmann (2007) argued that unbalanced bilinguals cannot completely suppress the L1 system or block its influence. In our study, most participants were proficient bilinguals. These results speak in favour of a model that suggests an interaction between the L1 and L2 systems across various levels, including the conceptual, phonological, and grammatical encoding levels, as stated in the Interactive Activation model of Dell (1986) or, the Independent Network model of Caramazza (1997). These models proposed that gender information in L2 is not stored as an inherent attribute of each lemma. Instead, it is dynamically calculated whenever needed, taking into account a comprehensive set of data, including information gleaned from the past linguistic inputs and the outcomes of competitive processes in activation. Consequently, accessing gender in L2 is not a straightforward or automatic consequence of selecting a word from the mental lexicon. It is, rather, a process that is contingent upon activation levels of both languages, perhaps, with the gender from L1 and the phonological form of the L2 word influencing the final selection of the form for production. In contrast, such models present a challenge for serial, modular models of speech production like the Levelt’s (1989) framework, which assumes that initial selection processes at the grammatical level are concluded before phonological encoding commences, making it implausible for the grammatical processes to affect the selection processes (e.g., gender determination) at the phonological level.

Second, external cues for gender information, like pictures, failed to facilitate processing L2 3rd person singular pronouns in proficient Chinese speakers of English. Thus, our main pattern of picture cues was different from Dong et al. (2015), who found a significant effect of gender saliency assisted by picture cues. This difference may be attributed to the differences of the tasks being used: Dong et al. (2015) tested their L2 speakers with a comprehension task (i.e., self-pace reading) and ours was a production task. These two tasks tap into different cognitive processes in language processing; therefore, the choice of a production task to simulate the real-life communication is more relevant to the problem we tried to solve.

Third, our results failed to demonstrate a default masculine gender use among Chinese-English speakers, which differed from the findings in Dong and Jia (2011) revealing that Chinese learners of English tended to err in the production of ‘SHE’ than ‘HE’. Their analysis of the Spoken English Corpus of Chinese Learners (SECCL), which comprises approximately 1.4 million words, showed an average error rate of 16.12% for feminine pronouns and 3.47% for masculine pronouns. SECCL was a corpus made up of spoken tests for Band 4 English majors in Chinese universities, whose proficiency level was moderate. Furthermore, in a specialized task involving Chinese-English interpretation by proficient learners, the error rate for feminine pronouns was significantly higher at 21.85%, while the rate for masculine pronouns was 7.92%. However, their analyses of *He-error* and *She-error* in both corpus and experiment studies were based on error rates per participant without considering each attempt/utterance of pronoun production at the word level. Subsequently, the statistical analyses were based on a ratio of accurate responses to the number of pronoun production, which can be misleading and losing lots of information, because both 1/1 and 5/5 yield the same 100%, despite the denominators. Therefore, a more accurate and appropriate analysis should be based on each attempt/utterance as a binary response (*he* or *she*) in logistic regression, as we did in our current report. Indeed, these two types of analyses generated different results in our current dataset, as the analysis based on error rates in ratio showed differences rising between *he* and *she* production while the current logistic analysis did not.

Nevertheless, our finding is consistent with that in Antón-Méndez (2010), which reported no difference between the error rates in *he* and *she* by Spanish learners of L2 English. As shown in 3.3, L2 proficiency did not significantly influence the overall production accuracy. This might suggest that high L2 proficiency results in insufficient variation to produce a detectable effect between *he* and *she* production, as our participants were highly proficient L2 speakers living overseas for an advanced academic degree.

Finally, AoA appeared to be a strong predictor in L2 gender production of 3rd person singular pronouns when there were no external cues, namely, the younger L2 speakers learned English the better performance was observed in the NoP condition, but no in the YesP condition. It could not be the case that these participants did not know that *he* denotes male antecedent and *she* female antecedent, as they must have learned this knowledge at the very early stage of L2 learning. The AoA effect is robust in L2 picture naming (e.g., Goggin et al., 1994). Grounded in connectionist modeling, Morrison et al. (2002) proposed that the AoA effects are thought to be situated in the connections between conceptual or semantic representations and their corresponding phonological representations. It is known that Chinese gender information relies solely on semantic cues while English has morphophonological cues together with semantic cues. The shift from semantic cues to morpho-phonological cues at an earlier age might help L2 production. It is posited that the connections for words acquired early are more deeply established (e.g., Zevin & Seidenberg, 2002), which implies that retrieving lexical forms from conceptual representations is more efficient and resilient for words learned early. According to this view, the influence of the AoA in advanced L2 speakers would stem from the stronger, more deeply ingrained connections between the conceptual/semantic representations and the phonological representations of the L2 words acquired early in life.

## Conclusion

Our findings reveal that 3rd person singular gender errors in L2 English learners are primarily a processing issue during speech production rather than a lack of grammatical knowledge. This suggests that learners may understand the English pronominal system (e.g., he vs. she) but struggle to apply it accurately in real-time production, especially under cognitive demands. Since this difficulty is influenced by L1 linguistic properties, explicit instruction alone may not suffice—learners need targeted processing practice to automatize correct form-meaning mappings.

Our findings provide pedagogical implications for second and foreign language teaching and learning. To enhance the accuracy of third-person singular pronoun production, materials and instructions must address the core challenge in learning: developing automatic form-meaning retrieval under communicative pressure while overcoming L1 transfer effects. This requires moving beyond traditional rule explanation towards a pedagogical framework that integrates four key principles. The foundation is consciousness-raising, which makes the L2 gender distinction salient through input flooding (Wu & Ionin, 2023). This awareness could then be operationalized through forced-attention processing practice, which demands real-time pronoun selection. This process can be further supported by visual scaffolding (Lestari & Misdi, 2018) to aid memory retrieval and solidified by consistent recasted feedback to reinforce correct form-meaning mappings without impeding communication.

The practical application of this framework leads to specific, evidence-based classroom techniques. Teachers can implement structured tasks—such as adapted story-retelling or “spot-the-pronoun” exercises—that compel learners to attend to gender agreement under time pressure. These activities should be designed with integrated visual aids, like character pictures or profiles, to provide a concrete referent in a discourse and strengthen the link between a referent and its pronoun. Crucially, during these practice sessions, instructors could provide implicit corrective feedback by recasting student errors, thus offering a correct model within the natural flow of discourse. This integrated approach ensures the practice mirrors the cognitive demands of real-world communication, fostering both accuracy and fluency in L2 speech.

While these findings may inform teaching practices, we should note that this study has certain limitations, particularly its small sample size (*n* = 32), which prevented subgroup analyses of the AoA and L2 proficiency effects. Future research with larger samples would allow for more comprehensive investigation of these factors.

## Supplementary Information

Below is the link to the electronic supplementary material.

Supplementary material 1 (DOCX 15.9 kb)

Supplementary material 2 (DOCX 25.1 kb)

Supplementary material 3 (PDF 316.5 kb)

## Data Availability

All supporting data were available at https://osf.io/zbjx7/?view_only=7d9a68961cc6407eb80982efc8d11a06.

## Footnotes

1 Dating back to the classical Chinese, 3rd person pronouns exhibited no gender distinction, as exemplified by the object pronoun 之 (zhī) and the possessive/attributive 其 (qí) (Wang, 1989; pp. 86–88). Prior to the 20th century, the character 他 (tā) served as a gender-neutral singular 3rd person pronoun encompassing “he”, “she”, and “it”. This system underwent significant modernization during the New Culture Movement (1915–1923) and May Fourth Movement (1919), when the gendered forms 她 (she) and 它 (it) were introduced under Western linguistic influence to correspond with Indo-European language conventions (Chan, 2011). In contemporary usage, 他 retains its default status for generic or gender-unspecified referents (e.g., “如果有人来, 让他等着”—“If someone comes, let them wait”), while 她 is employed only when female specificity requires emphasis, such as in feminist discourse or biographical contexts. This linguistic feature may lead Chinese learners of English to transfer the unmarked status of 他 to their L2 production, potentially resulting in the default use of masculine pronouns (e.g., “Everyone should bring his book”) even when context demands gender specificity.

2 Gender processing driven by grammar and pronoun might bear different mechanisms, i.e., at different stages based on Levelt’s production model. For example, gender processing for nouns may involve more complex grammatical encoding than pronoun gender systems. Though we reviewed the literature relevant to grammatical noun gender, we primarily want to show the evidence of the cross-language influence in gender processing.
