# Supplementary material for: She or He? Source of Errors in L2 Production of 3rd Person Singular Pronouns by Chinese Speakers of English
Source: J Psycholinguist Res. 2026 Apr 4;55(3):38. doi: 10.1007/s10936-026-10226-z (PMC13050342; doi:10.1007/s10936-026-10226-z)
Supplement: Supplementary file 1 — Supplementary material 1 (DOCX 15.9 kb) [file 10936_2026_10226_MOESM1_ESM.docx]

**Appendix A: Language History Questionnaire**

Name_____________ Age___________ Gender M / F

Email address_______________ Contact number_______________

**Part I: Non-school experience with English language**

1. Place of birth: ____________
2. Place(s) where you grew up: ________________

- If overseas(outside mainland China), please specify:

| ***Places*** | ***From what age and for how long*** |
| --- | --- |
|  |  |
|  |  |
|  |  |

1. Years and months in the UK ______________
2. Years and months in other English-speaking countries ______________
3. What language do you speak to your parents (dialects?)?

Mother:___________ Father:___________ Sibling(s):_________

1. At what age did you start speaking English? _____________

**Part II: School experience with English language**

1. Did you learn it in school (Formal education)? If yes, how many years have you learned English at school? ____________
2. Which programme(s) are you currently reading for at Oxford: ________________

**Part III: Language proficiency**

1. Please list all the languages you learned and rank them in terms of the criteria listed.

(1-Very poor; 2-Poor; 3-Fair; 4-Functional; 5-Good; 6-Very good; 7-Native-like)

| ***Language*** | ***Listening*** | ***Speaking*** | ***Reading*** | ***Writing*** | ***Use in the past six months (1- almost never; 2- occasionally; 3- often; 4- almost always)*** |
| --- | --- | --- | --- | --- | --- |
|  |  |  |  |  |  |
|  |  |  |  |  |  |
|  |  |  |  |  |  |

1. Your most recent IELTS score (if you have taken any)_______
2. Your most recent TOEFL score (if you have taken any)_______
