# Supplementary material for: She or He? Source of Errors in L2 Production of 3rd Person Singular Pronouns by Chinese Speakers of English
Source: J Psycholinguist Res. 2026 Apr 4;55(3):38. doi: 10.1007/s10936-026-10226-z (PMC13050342; doi:10.1007/s10936-026-10226-z)
Supplement: Supplementary file 2 — Supplementary material 2 (DOCX 25.1 kb) [file 10936_2026_10226_MOESM2_ESM.docx]

**Appendix B: Script of the Experiment Stimuli**

| Story No. | FF | MM | MIX | NoP |
| --- | --- | --- | --- | --- |
| **S1** | Lucy and mom  “I got up very early this morning. Then, my mom took me out for a walk. We saw a squirrel on the way back. | Tom and father  “I got up very early this morning. Then, my father took me out for a walk. We saw a squirrel on the way back. | FM: Lucy and father  “I got up very early this morning. Then, my father took me out for a walk. We saw a squirrel on the way back. | FF  Lucy and mom  “I got up very early this morning. Then, my mom took me out for a walk. We saw a squirrel on the way back. |
| S2 | Jenny and mother  “My mother bought me a Barbie for my birthday. I was so happy. I kissed my mom and said, ‘I love you, mom.’” | Jim and father  “My father bought me a model plane for my birthday. I was so happy. I kissed my daddy and said, ‘I love you, daddy.’” | FM: Jenny and father  “My father bought me a Barbie for my birthday. I was so happy. I kissed my daddy and said, ‘I love you, daddy.’” | MM  Jim and father  “My father bought me a model plane for my birthday. I was so happy. I kissed my daddy and said, ‘I love you, daddy.’” |
| S3 | Lily and friend  “I went to a movie with my friend, but we didn’t enjoy it. Now I’m annoyed because I paid $50 for the tickets.” | James and friend  “I went to a football match with my friend, but we didn’t enjoy it. Now I’m annoyed because I paid $50 for the tickets.” | FM:Lily and boyfriend  “I went to a movie with my boyfriend, but we didn’t enjoy it. Now I’m annoyed because I paid $50 for the tickets.” | FF  Lily and friend  “I went to a movie with my friend, but we didn’t enjoy it. Now I’m annoyed because I paid $50 for the tickets.” |
| S4 | Mrs. Green and daughter  “I worry that the violence in the games could cause my daughter to become violent. But my daughter doesn’t agree with me.” | Mr. Green and son  “I worry that the violence in the games could cause my son to become violent. But my son doesn't agree with me.” | FM: Mrs. Green and son  I worry that the violence in the games could cause my son to become violent. But my son doesn't agree with me.” | MM  Mr. Green and son  “I worry that the violence in the games could cause my son to become violent. But my son doesn't agree with me.” |
| S5 | Tina and aunt  Of all my relatives, I like my Aunt the best. She is of a similar age to me and often tells me interesting stories. | Harry and grandpa  Of all my relatives, I like my grandpa the best. He is the oldest in the family and often tells me interesting stories. | MF: Harry and grandmother  Of all my relatives, I like my grandmother the best. She is the oldest in the family and often tells me interesting stories. | FF  Tina and aunt  Of all my relatives, I like my Aunt the best. She is of a similar age to me and often tells me interesting stories. |
| S6 | Jessica and Anna  Anna is my best friend at school. I’d like to invite Anna to my home for a Sunday roast.I’m looking forward to that. | Jack and George  George is my best friend at school. I’d like to invite George to my home for a Sunday roast.I’m looking forward to that. | MF:Jack and Anna  Anna is my best friend at school. I’d like to invite Anna to my home for a Sunday roast.I’m looking forward to that. | MM  Jack and George  George is my best friend at school. I’d like to invite George to my home for a Sunday roast.I’m looking forward to that. |
| S7 | Emily and mom  I’m extremely bored, soI want to do something exciting for the weekend.I hope mom can take me to an amusement park. | Thomas and daddy  I’m extremely bored, soI want to do something exciting for the weekend.I hope Daddy can take me to an amusement park. | MF: Thomas and mom  I’m extremely bored, soI want to do something exciting for the weekend.I hope mom can take me to an amusement park. | FF  Emily and mom  I’m extremely bored, soI want to do something exciting for the weekend.I hope mom can take me to an amusement park. |
| S8 | Sophie and mother  I finished all the courses for my degree. My mother is very proud of me. She’s going to host a party to celebrate with me. | William and father  I finished all the courses for my degree. My father is very proud of me. He’s going to host a party to celebrate with me. | MF: William and wife  I finished all the courses for my degree. My wife is very proud of me. She’s going to host a party to celebrate with me. | MM  William and father  I finished all the courses for my degree. My father is very proud of me. He’s going to host a party to celebrate with me. |
| **S9** | Sophia and sister  I went to the space museum with my sister last year. Now I’d like to go to the Water World. What bus can take me there? | Sam and brother  I went to the space museum with my brother last year. Now I’d like to go to the Water World. What bus can take me there? | FM: Sophia and brother  I went to the space museum with my brother last year. Now I’d like to go to the Water World. What bus can take me there? | FF  Sophia and sister  I went to the space museum with my sister last year. Now I’d like to go to the Water World. What bus can take me there? |
| S10 | Phoebe and teacher  I really enjoy the English class given by Miss. LEE. This teacher is a graduate from Oxford. Miss. LEE’s class inspires me a lot! | Charlie and teacher  I really enjoy the English class given by Mr. LEE. This teacher is a graduate from Oxford. Mr. LEE’s class inspires me a lot! | FM: Isabella and teacher  I really enjoy the English class given by Mr. LEE. This teacher is a graduate from Oxford. Mr. LEE’s class inspires me a lot! | MM  Charlie and teacher  I really enjoy the English class given by Mr. LEE. This teacher is a graduate from Oxford. Mr. LEE’s class inspires me a lot! |
| S11 | Maggie and Miss Young  Miss Young teaches me English at school. I have some difficulty with pronunciation, so I often go to Miss Young’s office for help after school.” | Adam and Mr. Young  Mr. Young teaches me English at school. I have some difficulty with pronunciation, so I often go to Mr. Young’s office for help after school.” | FM: Maggie and Mr.Young  Mr. Young teaches me English at school. I have some difficulty with pronunciation, so I often go to Mr. Young’s office for help after school.” | FF  Maggie and Miss Young  Miss Young teaches me English at school. I have some difficulty with pronunciation, so I often go to Miss Young’s office for help after school.” |
| S12 | Julia and Emily  Emily offered to mend my computer. But she wanted £50 for helping me out! I couldn't afford it. | Robert and Tom  Tom offered to mend my computer. But he wanted £50 for helping me out! I couldn't afford it. | FM: Julia and Tom  Tom offered to mend my computer. But he wanted £50 for helping me out! I couldn't afford it. | MM  Robert and Tom  Tom offered to mend my computer. But he wanted £50 for helping me out! I couldn't afford it. |
| S13 | Emma and Tina  All my money disappeared. I don’t know what happened. Tina advised me to stop using eBay. | Henry and James  All my money disappeared. I don’t know what happened. James advised me to stop using eBay. | MF: Henry and Tina  All my money disappeared. I don’t know what happened. Tina advised me to stop using eBay. | FF  Emma and Tina  All my money disappeared. I don’t know what happened. Tina advised me to stop using eBay. |
| S14 | Alice and Jenny  Every night, I watch stars from my window with Jenny. I wish someone could get one for me. | David and Bob  Every night, I watch stars from my window with Bob. I wish someone could get one for me. | MF: David and sister  Every night, I watch stars from my window with my sister. I wish someone could get one for me. | MM  David and Bob  Every night, I watch stars from my window with Bob. I wish someone could get one for me. |
| S15 | Amy and Grace  “My sister Grace is 5 years older than me. Grace takes good care of me when my parents are not at home.” | John and James  My brother James is 5 years older than me. James takes good care of me when my parents are not at home.” | MF: John and Amy  My sister Amy is 5 years older than me. Amy takes good care of me when my parents are not at home.” | FF  Amy and Grace  “My sister Grace is 5 years older than me. Grace takes good care of me when my parents are not at home.” |
| S16 | Mrs. Brown and niece  I’m so fed up! I lost my cellphone yesterday! All my contacts are lost, including the number of my niece! | Mr. Brown and nephew  I’m so fed up! I lost my cellphone yesterday! All my contacts were lost, including the number of my nephew! | MF: Mr. Brown and niece  I’m so fed up! I lost my cellphone yesterday! All my contacts are lost, including the number of my niece! | MM  Mr. Brown and nephew  I’m so fed up! I lost my cellphone yesterday! All my contacts were lost, including the number of my nephew! |
| **S17** | Rose and aunt  I got lost in the city centre. Fortunately, my aunt happened to see me and kindly drove me home. | Owen and uncle  I got lost in the city centre. Fortunately, my uncle happened to see me and kindly drove me home. | FM: Rose and uncle  I got lost in the city centre. Fortunately, my uncle happened to see me and kindly drove me home. | FF  Rose and aunt  I got lost in the city centre. Fortunately, my aunt happened to see me and kindly drove me home. |
| S18 | Ella and boss  My boss pushes me so much that I have been working overtime for 2 weeks! I simply have no time left for my family. | Charles and boss  My boss pushes me so much that I have been working overtime for 2 weeks! I simply have no time left for my family. | FM: Ella and boss  My boss pushes me so much that I have been working overtime for 2 weeks! I simply have no time left for my family. | MM  Charles and boss  My boss pushes me so much that I have been working overtime for 2 weeks! I simply have no time left for my family. |
| S19 | Miss Wright and manager  I didn’t get the job I applied for.The manager told me that I need more experience, though my degree is very good. | Mr. Wright and manager  I didn’t get the job I applied for.The manager told me that I need more experience, though my degree is very good. | FM: Miss Wright and manager  I didn’t get the job I applied for.The manager told me that I need more experience, though my degree is very good. | FF  Miss Wright and manager  I didn’t get the job I applied for.The manager told me that I need more experience, though my degree is very good. |
| S20 | Hanna and grandma  When I was 5 years old, my grandma gave me a violin. I took my violin with me everywhere. | Bill and grandpa  When I was 10 years old, my grandpa gave me a laptop. I took my laptop with me everywhere. | FM: Hanna and grandpa  When I was 5 years old, my grandpa gave me a violin. I took my violin with me everywhere. | MM  Bill and grandpa  When I was 10 years old, my grandpa gave me a laptop. I took my laptop with me everywhere. |
| S21 | Anna and roommate  I chatted with my roommate until 2 am, so I was too tired today in the exam. I couldn’t read the questions. | Michael and roommate  I watched a football match with my roommate until 2 am, so I was too tired today in the exam. I couldn’t read the questions. | MF: Michael and sister  I watched a football match with my sister until 2 am, so I was too tired today in the exam. I couldn’t read the questions. | FF  Anna and roommate  I chatted with my roommate until 2 am, so I was too tired today in the exam. I couldn’t read the questions. |
| S22 | Eva and Emilie  I went to Oxford and studied law. My cousin Emilie went to a fashion school in London. Four years later, I’m still a poor student, but my cousin is rich and famous. | Daniel and Jim  I went to Oxford and studied law. My cousin Jimwent to a fashion school in London. Four years later, I’m still a poor student, but my cousin is rich and famous. | MF: Daniel and Emily  I went to Oxford and studied law. My cousin Emily went to a fashion school in London. Four years later, I’m still a poor student, but my cousin is rich and famous. | MM  Daniel and Jim  I went to Oxford and studied law. My cousin Jimwent to a fashion school in London. Four years later, I’m still a poor student, but my cousin is rich and famous. |
| S23 | Mrs. Wood and daughter  My daughter Alice often uses my computer and I’m pretty sure that Alice knows my password. I have to update it. | Mr. Wood and son  My son John often uses the computer and I’m pretty sure that John knows my password. I have to update it. | MF: Mr. Wood and daughter  My daughter Alice often uses my computer and I’m pretty sure that Alice knows my password. I have to update it. | FF  Mrs. Wood and daughter  My daughter Alice often uses my computer and I’m pretty sure that Alice knows my password. I have to update it. |
| S24 | Grace and Lucy  I don’t like red wine. I’d like a glass of dry white. Is that okay for you, Lucy? | Harry and George  I don’t like red wine. I’d like a glass of dry white. Is that okay for you, George? | MF: Harry and Lucy  I don’t like red wine. I’d like a glass of dry white. Is that okay for you, Lucy? | MM  Harry and George  I don’t like red wine. I’d like a glass of dry white. Is that okay for you, George? |
| S25 | Ellie and Lola  I have had pain in my knees for 2 weeks, so Lola advised me to go to the hospital. I hope there’s no problem with my knees. | Alexander and James:  I have had pain in my knees for 2 weeks, so James advised me to go to the hospital. I hope there’s no problem with my knees. | MF: Alexander and Lucy  I have had pain in my knees for 2 weeks, so Lucy advised me to go to the hospital. I hope there’s no problem with my knees. | FF: Ellie and Lola  I have had pain in my knees for 2 weeks, so Lola advised me to go to the hospital. I hope there’s no problem with my knees. |
| S26 | Isabel and friend  I got the time wrong and I missed my plane. My friend told me it’s not the end of the world. | Jack and friend  I got the time wrong and I missed my plane. My friend told me it’s not the end of the world. | MF: Jack and cousin  I got the time wrong and I missed my plane. My cousin told me it’s not the end of the world. | MM: Jack and friend  I got the time wrong and I missed my plane. My friend told me it’s not the end of the world. |
| S27 | Bella and Elizabeth  My teacher Elizabeth had a problem with the computer, and asked me to look at it. Unfortunately, I’m not an expert. | Adam and teacher  My teacher had a problem with the computer, and asked me to look at it. Unfortunately, I’m not an expert. | MF: Adam and teacher  My teacher had a problem with the computer, and asked me to look at it. Unfortunately, I’m not an expert。 | FF: Bella and Elizabeth  My teacher Elizabeth had a problem with the computer, and asked me to look at it. Unfortunately, I’m not an expert. |
| S28 | Evelyn and roommate  I usually spend hours playing online games. My roommate tells me that it’s bad for my health. | Arthur and father  I usually spend hours in my room playing online games. My father tells me that it’s bad for my health. | MF: Arthur and mother  I usually spend hours in my room playing online games. My mother tells me that it’s bad for my health. | MM: Arthur and father  I usually spend hours in my room playing online games. My father tells me that it’s bad for my health. |
| S29 | Sarah and Sophie  My friend Sophie will get married next month. I’m very excited. Sophie has sent me an invitation for the wedding. | Ben and Peter  My friend Peter will get married next month. I’m very excited. Peter has sent me an invitation for the wedding. | FM: Sarah and Peter  My friend Peter will get married next month. I’m very excited. Peter has sent me an invitation for the wedding. | FF: Sarah and Sophie  My friend Sophie will get married next month. I’m very excited. Sophie has sent me an invitation for the wedding. |
| S30 | Evie and mom  I can’t find my scarf. I think maybe I’ve forgotten it in my bedroom. Can you check it for me, mom? | Alex and dad  I can’t find my scarf. I think maybe I’ve forgotten it in my bedroom. Can you check it for me, dad? | FM: Evie and dad  I can’t find my scarf. I think maybe I’ve forgotten it in my bedroom. Can you check it for me, dad? | MM: Alex and dad  I can’t find my scarf. I think maybe I’ve forgotten it in my bedroom. Can you check it for me, dad? |
| S31 | Daisy and grandma  My grandma died when I was six. She left me thousands of books. | Lucas and grandpa  My grandpa died when I was six. He left me thousands of books. | FM: Daisy and grandpa  My grandpa died when I was six. He left me thousands of books. | FF: Daisy and grandma  My grandma died when I was six. She left me thousands of books. |
| S32 | Isabelle and Phoebe  I had a big party last night. Unfortunately, my head hit the corner of my bed and started bleeding. My friend Phoebe took me to the hospital. | Oscar and flatmate  I had a big party last night. Unfortunately, my head hit the corner of my bed and started bleeding. My flat mate took me to the hospital. | FM: Isabelle and Sam  I had a big party last night. Unfortunately, my head hit the corner of my bed and started bleeding. My flat mate Sam took me to the hospital. | MM: Oscar and flatmate  I had a big party last night. Unfortunately, my head hit the corner of my bed and started bleeding. My flat mate took me to the hospital. |
