# Supplementary material for: She or He? Source of Errors in L2 Production of 3rd Person Singular Pronouns by Chinese Speakers of English
Source: J Psycholinguist Res. 2026 Apr 4;55(3):38. doi: 10.1007/s10936-026-10226-z (PMC13050342; doi:10.1007/s10936-026-10226-z)
Supplement: Supplementary file 3 — Supplementary material 3 (PDF 316.5 kb) [file 10936_2026_10226_MOESM3_ESM.pdf]

Relative Importance Analysis

server.R ui.R ↴ show below

Raw Data | Correlation Matrix | About

Using Raw Data

Note: Input values must be separated by tabs. Copy and paste from Excel/Numbers.

Wait patiently. It will take a minute or so until you get to see the result.
Please make sure that your data includes the header (variable names) in the first row.
The criterion (dependent) variable should be placed in the first column.

```
 6  0.925925926 0.203582838 1.50507541
 7  0.863636364 0.946061425 -0.641507552
 8  1   0.203582838 0.074020102
 9  0.96969697  0.203582838 -1.357035206
10  1   -0.538895748    2.220603064
11  1   -1.281374335    -0.641507552
12  0.772727273 -0.538895748    -1.357035206
13  1   -1.281374335    0.789547756
14  0.916666667 -0.538895748    0.789547756
15  1   -1.281374335    0.074020102
16  0.931034483 -0.167656455    0.789547756
17  0.961538462 -0.538895748    -2.072562859
18  0.96    0.946061425 0.074020102
19  1   -0.538895748    0.074020102
20  1   -1.281374335    0.074020102
21  1   -1.652613628    0.789547756
22  0.842105263 0.946061425 0.074020102
23  1   0.574822132 0.789547756
24  0.72    1.317300718 0.074020102
25  0.9 1.317300718 -0.641507552
26  1   -0.167656455    -0.641507552
27  0.818181818 0.946061425 -1.357035206
28  0.95    1.317300718 -2.072562859
29  0.956521739 -0.538895748    0.074020102
30  0.90625 0.946061425 0.074020102
```

Run Analysis

Basic Statistics

```
            n  mean   sd median trimmed  mad   min  max range  skew kurtosis
ACC        29  0.94 0.08   0.96    0.95 0.06  0.72 1.00  0.28 -1.18     0.31
zAoA       29 -0.05 1.00  -0.17    0.00 1.65 -2.40 1.32  3.71 -0.37    -0.85
zTestscore 29  0.00 1.00   0.07    0.02 1.06 -2.07 2.22  4.29 -0.28    -0.23
             se
ACC        0.01
zAoA       0.19
zTestscore 0.19
```

Correlation

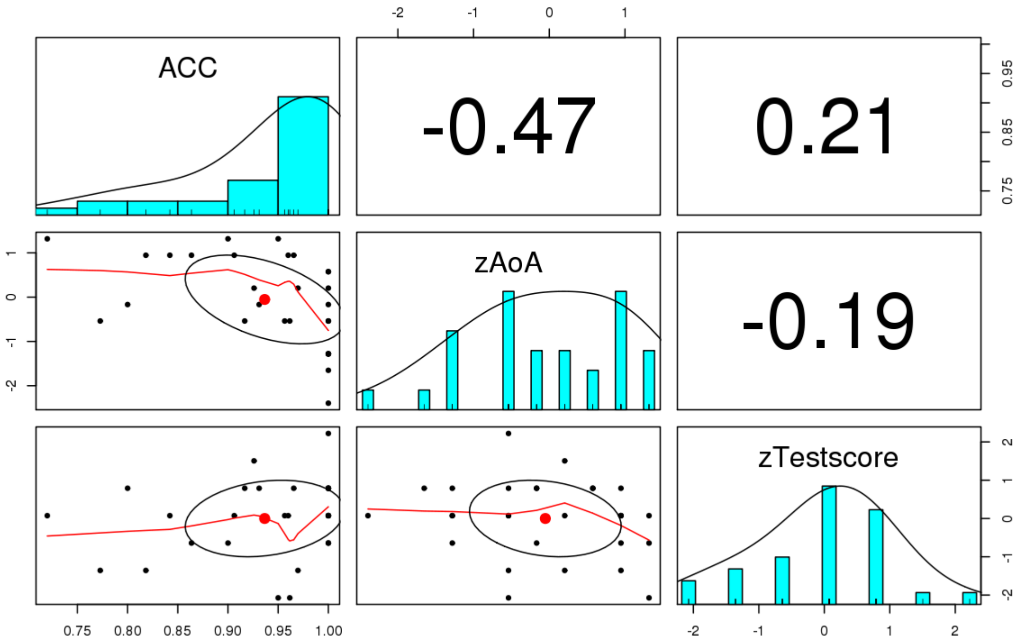


```
library(shiny)
library(shinyAce)
library(psych)
library(car)
library(rpsychi)
library(boot)
library(plyr)
library(ggplot2)
library(Boruta)
library(relaimpo)
library(MASS)



shinyServer(function(input, outp
ut, session) {

    observe({
        if (input$do > 0) {

#--------------------------------
--------------------------------
--
# Relative Weight Analysis Using
Raw Data
#--------------------------------
--------------------------------
--

            # Basic statistics
                bs <- reactive({
                    x <- read.cs
v(text=input$text, sep="\t")
                    describe(x)
[2:13]
                })

                output$textarea.
out <- renderPrint({
                    bs()
                })


            # Correlation
                makecorPlot <- f
unction(){
                    x <- read.cs
v(text=input$text, sep="\t")
                    pairs.panels
(x)
                }

                output$corPlot <
- renderPlot({
                    print(makeco
rPlot())
                })


            # Regression
                reg <- reactive
({
                    [illegible]
```

Code license: GPL-3 (https://gnu.org/licenses/gpl-3.0.txt)

Regression Analysis

```

Call:
lm(formula = Criterion ~ ., data = dat)

Residuals:
     Min       1Q   Median       3Q      Max 
-0.16943 -0.01914  0.01779  0.05579  0.08223 

Coefficients:
            Estimate Std. Error t value Pr(>|t|)    
(Intercept)  0.93471    0.01337  69.894   <2e-16 ***
zAoA        -0.03494    0.01381  -2.531   0.0178 *  
zTestscore   0.01009    0.01384   0.729   0.4725    
---
Signif. codes:  0 ‘***’ 0.001 ‘**’ 0.01 ‘*’ 0.05 ‘.’ 0.1 ‘ ’ 1

Residual standard error: 0.07191 on 26 degrees of freedom
Multiple R-squared:  0.2335,	Adjusted R-squared:  0.1746 
F-statistic: 3.961 on 2 and 26 DF,  p-value: 0.0315



 ---
 Standardized beta estimates:
           Standardized beta
zAoA                  -0.443
zTestscore             0.128

 ---
 VIF and tolerance statistic (1/VIF):
             VIF Tolerance
zAoA       1.037     0.964
zTestscore 1.037     0.964

 VIF should be smaller than 10 (clozer to 1 better);
 tolerance statistic (1/VIF) should be greater than 0.2.
```

Dominance Analysis

```
           Weight
zAoA       0.2033
zTestscore 0.0302
```

Relative Weight Analysis

```
R-squared For the Model:
[1] 0.2335414

 The Raw and Rescaled Weights:
   Variables Raw.RelWeight Rescaled.RelWeight
1       zAoA    0.20332956           87.06361
2 zTestscore    0.03021184           12.93639

 BCa Confidence Intervals around the raw weights:
   Variables CI.Lower.Bound CI.Upper.Bound
1       zAoA   0.0101018271      0.4365827
2 zTestscore   0.0008241519      0.2229991

 BCa Confidence Interval Tests of significance:
  (If 0 is not included, weight is significant at p < .05)
      Labels CI.Lower.Bound CI.Upper.Bound
1       zAoA    -0.02493183      0.4683263
2 zTestscore    -0.08854512      0.2401826
```

Relative Weight Plot with 95% CI

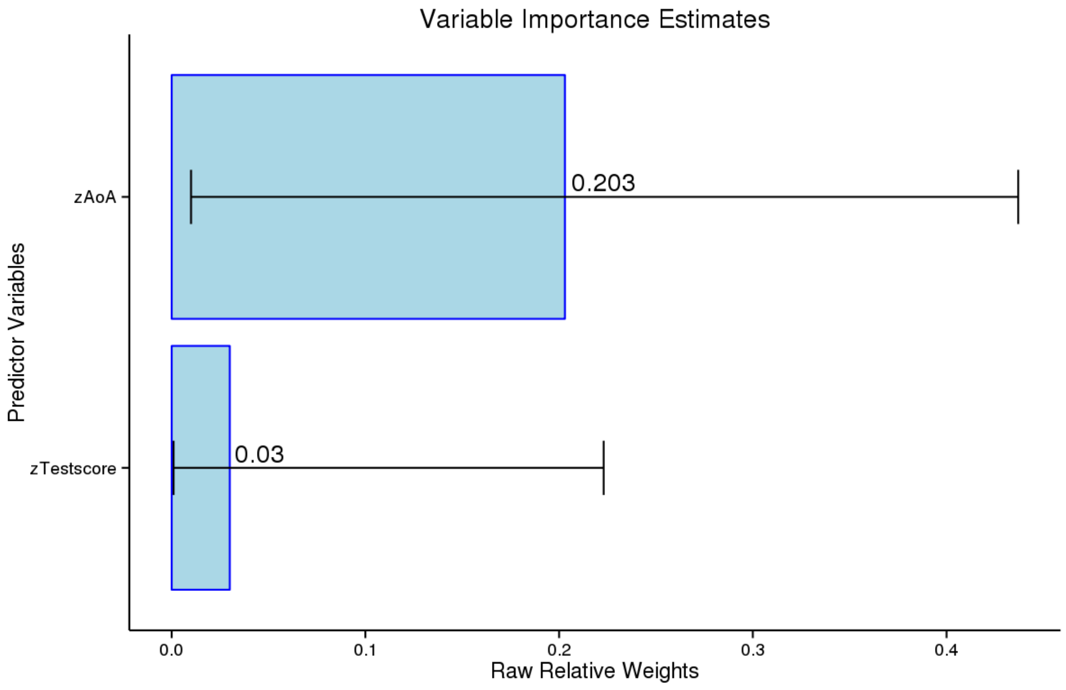

Feature Selection: Variable Importance (Random Forest)

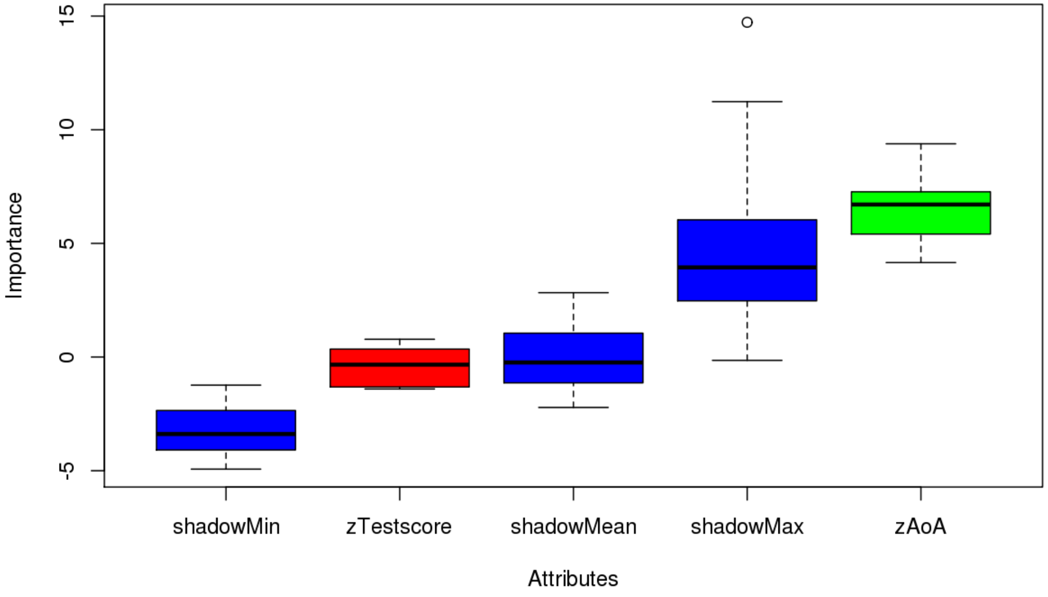


```
[[1]]
Boruta performed 26 iterations in 0.4944952 secs.
 1 attributes confirmed important: zAoA.
 1 attributes confirmed unimportant: zTestscore.

[[2]]
                meanZ   medianZ      minZ      maxZ  normHits  decision
zAoA        6.5372830  6.710749  4.155880 9.3823203 0.7692308 Confirmed
zTestscore -0.4031537 -0.334095 -1.403668 0.7820829 0.0000000  Rejected
```

R session info

```
This analysis was conducted with R version 3.2.1.
It was executed on Thu Nov 14 21:31:10 2024.
```

About

You can check out this application here: - http://langtest.jp/shiny/rwa/

Author

Atsushi MIZUMOTO (http://mizumot.com/), Ph.D.
Associate Professor of Applied Linguistics
Faculty of Foreign Language Studies, Kansai University, Osaka, Japan
